# Association between periodontitis and breast cancer in Indian women – a pilot study in Dakshina Kannada, Karnataka

**DOI:** 10.1186/s12903-025-06773-x

**Published:** 2025-10-03

**Authors:** Aparna K. S, Avinash B. R, Rajesh Gururaghavendran

**Affiliations:** 1 Centre for Dental Education and Research, All India Institute of Medical Sciences, New Delhi, India; 2 Department of Public Health Dentistry, Manipal College of Dental Sciences Mangalore, Manipal Academy of Higher Education, Manipal, India; 3 Department of Health Outcomes and Biomedical Informatics, Institute for Child Health Policy, College of Medicine, University of Florida, 2197 Mowry Road, Gainesville, FL 32611 USA

**Keywords:** Breast cancer, Chronic inflammation, Oral health, Periodontitis, Risk factors, Women health

## Abstract

**Background:**

The most prevalent cancer in women is breast cancer (BC), and its prevalence is on the rise in India. Chronic inflammatory diseases like periodontitis have been connected to the development of cancer and systemic inflammation. This study aimed to assess the relationship between periodontitis and breast cancer in Indian women.

**Methods:**

This cross-sectional pilot study involved 60 women from a tertiary hospital in Mangalore, Karnataka (30 BC patients and 30 age and use of tobacco matched controls). Clinical exams, standardized questionnaires, and medical records were used to gather data. The WHO Oral Health Assessment Form (2013) was used to evaluate periodontal health. After controlling for variables, the relationship between periodontitis and breast cancer was assessed using multivariate logistic regression.

**Results:**

The periodontal health of BC patients was considerably worse when compare to controls. as They had higher levels of gingival recession (*p* = 0.03), clinical attachment loss (*p* = 0.02), bleeding on probing (*p* = 0.02), probing depth (*p* = 0.04), plaque index (*p* = 0.03), and gingival index (*p* = 0.04). The risk for breast cancer was 2.79 times higher in those with moderate periodontitis (aOR = 2.79, *p* = 0.002) and 4.27 times higher in those with severe periodontitis (aOR = 4.27, *p* < 0.001). Generalized periodontitis was significantly associated with breast cancer compared to localized periodontitis(aOR = 2.25,*p* = 0.004).

**Conclusion:**

There is a significant association between periodontitis and breast cancer. The possibility that chronic periodontal inflammation increases the risk of breast cancer highlights the need for more studies and integrated healthcare strategies.

## Introduction

Breast cancer (BC) is the most prevalent malignancy among women worldwide [1]. Data from the International Agency for Research on Cancer (IARC) registry suggest that 45% of newly diagnosed cases of breast cancer and 55% of breast cancer related mortality currently occur in low and middle-income countries like India [2]. The rising incidence rate among women can be attributed to earlier menarche, later first pregnancy, fewer pregnancies, shorter breastfeeding periods, and later menopause. Obesity, alcohol consumption, and a lack of physical activity are all rising risk factors for breast cancer [3]. Owing to the lack of awareness of this disease and in absence of a breast cancer screening program, the majority of breast cancers are diagnosed at a relatively advanced stage [2]. 

Periodontitis is a chronic, complex inflammatory disease caused by plaque biofilm that causes irreversible and damaging immune responses. Periodontitis affects 45–50% of the global population, with severe cases affecting 11.2% of the population and ranking as the sixth most frequent human disease [4]. According to Janakiram et al. [5] the prevalence of periodontal diseases is 51% in adults in India.

Periodontal disease conditions are known to be associated with chronic systemic inflammatory reactions like increased blood C-reactive protein, cytokines and chemokines, which play a major role in impacting carcinogenesis. Bacterial toxins and biproducts produced in the mouth like nitrosamines and acetaldehyde could have a systemic impact on carcinogenesis. It may be aggravated by common risk factors such as smoking, physical activities, dietary habits as well as etiologic factors such as inflammation, oxidative stress, or genetics. The cytokines released during periodontitis may add to breast cancer and metastasis [6]. 

Several research have looked into the potential link between periodontitis and breast cancer. Camilo et al. [3] conducted a hospital-based case-control study in Brazil with 67 breast cancer cases and 134 controls and found that clinical attachment loss was significantly higher in breast cancer patients and periodontitis increased breast cancer risk by 2–3 times. Freudenheim et al. [6] studied postmenopausal women and discovered that those with self-reported periodontal disease had a 14% higher breast cancer risk, with the strongest association in former smokers who had quit within 20 years. Soder et al. [7] found a substantial link between periodontal disease/tooth loss and breast cancer. In contrast, Mai et al. [8] found no link between periodontal infections and breast cancer in postmenopausal women.

Differences in study design, adjustment for variables, periodontal evaluation, and limited sample size may contribute to inconsistent findings in breast cancer research. The evidence supporting the link between periodontitis and breast cancer is limited and contradictory. Examining this potential association can yield significant insights for public health practitioners, considering the recurrent occurrences of many oral health problems. If a link exists, such findings could contribute to breast cancer prevention strategies. The aim of this study was to ascertain the relationship between periodontitis and breast cancer among study participants.

## Materials and methods

### Study design

This pilot study employed an analytical cross-sectional study design. The study was carried out in compliance with the Declaration of Helsinki and received ethical approval from the Institutional Ethics committee of Manipal College of Dental Sciences, Mangalore (Ref. no: 18084). Permission to carry out the present study was obtained from the hospital authorities. Informed consent was obtained from the study subjects.

## Participants and sampling

The study was conducted among 60 participants (30 cases, 30 controls) in Mangalore, Karnataka between September 2021 to January 2022.

A sample size of 30 cases and 30 matched controls was chosen for the pilot study in accordance with accepted guidelines for feasibility studies. While Hertzog [9] suggests that 30 participants per group are adequate to evaluate variability and test study methods, Lackey and Wingate [10] propose that pilot studies may involve approximately 10% of the sample needed for a full-scale study. This size also balances logistical feasibility with the ability to explore preliminary associations. Although not powered for definitive conclusions, the chosen sample is appropriate for the study’s exploratory purpose and consistent with pilot study norms.

Participants were the female patients referred to Department of Oncology diagnosed as having breast cancer in a tertiary hospital in Mangalore. Age, gender and use of tobacco matched controls without breast cancer was included in the present study. The controls were recruited from the bystanders accompanying the patients in the hospital with self-reported negative cancer history. The study subjects who satisfied the inclusion and exclusion criteria for the study were recruited. A convenience sampling method was employed due to logistical constraints and limited access to eligible subjects within the hospital setting. The use of convenience sampling is acknowledged as a limitation of the study and may introduce potential selection bias.

Dentate women ≥ 18 years diagnosed with breast cancer (irrespective of the cancer staging and treatment), those who were deemed to be in remission or who had finished cancer treatment were included. Women without a history of breast cancer or any other cancer who were accompanying the patients were chosen as controls. Women with an ICD-10 D05 diagnosis of in situ breast cancer, those having a diagnosis of psychomotor disorders, those using fixed orthodontic appliances, those taking drugs linked to gingival overgrowth (such as cyclosporine, phenytoin, and nifedipine), or those who need antibacterial prophylaxis before oral exams were excluded.

The investigators (A.K.S. and A. B. R) were trained and calibrated prior to start of the study to ensure reliability (k = 0.80). Data were collected using a structured questionnaire followed by clinical examination by the two calibrated investigators and recorded by a trained assistant. Details regarding breast cancer history and treatment were obtained from the hospital records. Body Mass Index (BMI) was calculated by measuring height and weight. The results were grouped according to WHO’s BMI classification as underweight, normal weight, overweight, and obese [11]. 

With the exception of third molars, a full-mouth periodontal clinical examination was performed at six different sites per tooth: buccal, distobuccal, mesiolingual, lingual, and distal-lingual. Clinical assessment of periodontal condition was done using WHO oral health assessment form 2013 for adults [12]. The Plaque Index (PII) [13], Gingival Index (GI) [14], Bleeding on probing (BoP), Probing Depth (PD), Clinical Attachment Loss (CAL) and Gingival Recession (GR) were recorded.

PPD was defined as “the distance from the free gingival margin to the bottom of the pocket or gingival sulcus”. CAL was defined as “the distance from the cementoenamel junction to the bottom of the pocket or gingival sulcus” [3]. Gingival recession is defined as “the displacement of the gingival margin apical to the cemento-enamel junction (CEJ) of a tooth.” [15].

A Community Periodontal Index of Treatment Needs (CPITN) probe was used to take measurements, which were rounded to the nearest whole millimeter. Alongside the PPD value, BoP was noted as either present or absent.

Breast cancer was identified as a dichotomous variable and was the study’s outcome. Periodontitis was the primary exposure variable, and it was divided into the following categories according to the severity. The general definition of extent was Localized = ≤ 30% of sites involved and Generalized = > 30% of sites included. Based on 1999 American Academy of Periodontology (AAP) Classification, the severity of periodontitis was determined using Clinical Attachment Loss (CAL) as follows:

(i) Mild periodontitis = 1 or 2 mm CAL
(ii) Moderate periodontitis = 3 or 4 mm CAL
(iii) Severe periodontitis = ≥ 5 mm CAL [16]

### Statistical analysis

Statistical analysis was done using IBM SPSS Statistics V22.0. Data was tested for normality and was found to be normally distributed. A chi-squared test and Fisher exact post hoc test (α = 0.05) was employed to compare the prevalence of breast cancer and periodontitis between Breast Cancer groups (BC Group) and control groups.

A multivariate analysis was conducted to assess the associations between breast cancer and periodontitis. Although temporality could not be established due to the cross-sectional design, potential confounding from treatment effects was addressed statistically. Participants were stratified by treatment status, and treatment-related variables (e.g., time since diagnosis, type of treatment) and were included as covariates in the multivariate analysis. The following potential confounders were adjusted for in the multivariate model: Age, Socioeconomic status (SES), Marital status, Cancer stage at diagnosis, Time since diagnosis, Treatment status, Surgery type, Chemotherapy, Radiotherapy, Last dental visit, BMI, Family history of breast cancer, Diabetes Mellitus, Bisphosphonate use, Smoking status, Alcohol use, Parity, Age at first birth, Total breastfeeding time, Oral Contraceptive use, Time since menopause, Hormone Replacement Therapy and Frequency of brushing. Adjusted odds ratios (aOR) with 95% confidence intervals (CIs) were reported. A *p*-value < 0.05 was considered statistically significant.

## Results

A total of 60 participants (30 cases, 30 controls) were recruited in the pilot study. Among the 30 women with breast cancer, 66.7% were diagnosed within the past 12 months, and 22 (73.3%) had early-stage disease (Stage I or II). Most had received chemotherapy (83.3%), radiotherapy (60.0%), and lumpectomy (73.3%), while 26.7% underwent mastectomy. Additionally, 20 (66.7%) were on hormonal therapy, and 01 (3.3%) had metastatic disease.

Participants in the breast cancer (BC) group were 50.7 ± 8.4 years old on average, while those in the control group were 49.5 ± 7.1 years old (*p* = 0.21). There was no significant difference between the groups in terms of SES, marital status, last dental visit, BMI classification, family history of breast cancer, diabetes, bisphosphonate use, smoking status, and alcohol use (*p* > 0.05). However, parity (*p* = 0.04), age at first birth (*p* = 0.02), duration of oral contraceptive usage (*p* = 0.03), total breastfeeding time (*p* = 0.01), and hormone replacement treatment (*p* = 0.04) all showed statistically significant differences. Having no children (23.3% vs. 6.7%, *p* = 0.04) and having their first child beyond the age of 30 (16.7% vs. 3.3%, *p* = 0.02) were significantly higher among BC group. They also had a higher prevalence of breastfeeding for less than 12 months (13.3% vs. 10.0%, *p* = 0.01). Both the use of hormone replacement therapy (26.7% vs. 16.7%, *p* = 0.04) and long term oral contraceptive use (≥ 240 months) were also greater in the breast cancer group (26.7% vs. 6.7%, *p* = 0.03). (Table 1).

**Table 1:** Demographic and lifestyle characteristics of study participants

Variables		Breast cancer group *N* = 30	Control group *N* = 30	*p* value
Age (years)	Mean ± S. D	50.7± 8.4	49.5± 7.1	0.21
Socioeconomic status (SES)	Upper (I)	01 (3.3)	02 (6.7)	0.43
	Upper middle (II)	06 (20.0)	04 (13.3)	
	Lower middle (III)	16 (53.4)	12 (40.0)	
	Upper lower (IV)	06 (20.0)	09 (30.0)	
	Lower (V)	01 (3.3)	03 (10.0)	
Marital status	Married	28 (93.3)	29 (96.7)	0.61
	Not Married	02 (6.7)	01 (3.3)	
Last dental visit	Within last 6 months	08 (26.7)	05 (16.7)	0.32
	More than 6 months	22 (73.3)	25 (83.3)	
BMI Classification	Underweight (< 18.5)	03 (10.0)	02 (6.7)	0.24
	Normal (18.5–24.9)	23 (76.6)	21 (70.0)	
	Overweight (25-29.9)	02 (6.7)	04 (13.3)	
	Obese (≥ 30)	02 (6.7)	03 (10.0)	
Family history of breast cancer	Yes	04 (13.3)	01 (3.3)	0.41
	No	26 (86.7)	29 (96.7)	
Diabetes	Yes	05 (16.7)	08 (26.7)	0.32
	No	25 (83.3)	22 (73.3)	
Bisphosphonate use	Yes	11 (63.3)	09 (30.0)	0.34
	No	19 (6.7)	21 (70.0)	
Smoking status	Current	02 (6.7)	02 (6.7)	0.51
	Past	01 (3.3)	04 (13.3)	
	Never	27 (90.0)	21 (70.0)	
Alcohol use	Yes	01 (3.3)	02 (6.7)	0.67
	No	29 (96.7)	28 (93.3)	
Parity	No children	07 (23.3)	02 (6.7)	0.04*
	1–2 children	22 (73.3)	24 (80.0)	
	≥3 children	01 (10.0)	04 (13.3)	
Age at first birth	No children	07 (23.3)	02 (6.7)	0.02*
	<20 years	02 (6.7)	03 (10.0)	
	>20-<30 years	16 (53.3)	24 (80.0)	
	≥ 30 years	05 (16.7)	01 (3.3)	
Total breastfeeding time	No children	01 (3.3)	02 (6.7)	0.01*
	<12 months	04 (13.3)	03 (10.0)	
	>12-<36 months	22 (73.4)	18 (60.0)	
	≥ 36 months	04 (13.3)	07 (23.3)	
Oral contraceptive use	No use	03 (10.0)	05 (16.7)	0.03*
	≥ 1-<120 months	11 (36.6)	13 (43.3)	
	>120-<240 months	08 (26.7)	10 (33.3)	
	≥ 240 months	08 (26.7)	02 (6.7)	
Time since menopause	No menopause	06 (20.0)	05 (16.7)	0.23
	≥ 1-<60 months	13 (43.4)	17 (56.6)	
	>60-<120 months	10 (33.3)	06 (6.7)	
	≥ 120 months	01 (3.3)	02 (25.0)	
Hormone replacement therapy	Yes	08 (26.7)	05 (16.7)	0.04*
	No	22 (73.3)	25 (83.3)	
Frequency of brushing	Everyday	25 (83.3)	23 (76.7)	0.49
	More than once a day	05 (16.7)	07 (23.3)	

Chi-square test of association

*Statistically significant at *p* < 0.05

In comparison to the control group, the BC group’s periodontal health measures showed a noticeably inferior state of periodontal health. The BC group had significantly higher mean plaque index (2.3 ± 0.6 vs. 1.4 ± 0.7, *p* = 0.03), gingival index (1.6 ± 0.2 vs. 1.1 ± 0.4, *p* = 0.04), and bleeding on probing (47.2 ± 9.7 vs. 34.3 ± 8.6, *p* = 0.02). Similarly, they had significantly higher gingival recession (1.2 ± 0.2 mm vs. 0.3 ± 0.6 mm, *p* = 0.03), clinical attachment loss (3.9 ± 0.9 mm vs. 2.7 ± 1.1 mm, *p* = 0.02), and probing depth (3.5 ± 0.7 mm vs. 2.6 ± 0.8 mm, *p* = 0.04). They also had significantly higher proportions of sites with gingival recession ≥ 1 mm (33.3% vs. 10.0%, *p* = 0.04), clinical attachment loss ≥ 3 mm (40.0% vs. 16.7%, *p* = 0.03), and probing depth ≥ 4 mm (26.7% vs. 3.3%, *p* = 0.02) (Table 2).

**Table 2:** Periodontal parameters among cases and controls

Variables	Breast cancer group *N* = 30	Control group *N* = 30	*p* value
Mean Number of Teeth	21.2 ± 2.3	23.1 ± 4.1	0.62
Mean Plaque Index	2.3 ± 0.6	1.4 ± 0.7	0.03*
Mean Gingival Index	1.6 ± 0.2	1.1 ± 0.4	0.04*
Mean Bleeding on Probing (BoP) (% Sites)	47.2 ± 9.7	34.3 ± 8.6	0.02*
Mean Probing Depth (PD) (mm)	3.5 ± 0.7	2.6 ± 0.8	0.04*
Mean Clinical Attachment Loss (CAL) (mm)	3.9 ± 0.9	2.7 ± 1.1	0.02*
Mean Gingival Recession (GR) (mm)	1.2 ± 0.2	0.3 ± 0.6	0.03*
Percentage of sites with PD ≥ 4 mm	08 (26.7)	01 (3.3)	0.02*
Percentage of sites with CAL ≥ 3 mm	12 (40.0)	05 (16.7)	0.03*
Percentage of sites with GR ≥ 1 mm	10 (33.3)	03 (10.0)	0.04*

Unpaired t-Test

*Statistically significant at *p* < 0.05

The multivariate logistic regression showed association between periodontitis and breast cancer remained substantial even after controlling for potential variables. The risk of breast cancer was significantly increased by moderate and severe periodontitis; the risk was increased by 2.79 times for moderate periodontitis (aOR = 2.79, 95% CI: 1.57–4.89, *p* = 0.002) and 4.27 times for severe periodontitis (95% CI: 2.31–8.26, *p* < 0.001), while mild periodontitis did not significantly increase the risk (aOR = 1.52, *p* = 0.16). Additionally, generalized periodontitis was significantly associated with breast cancer compared to localized periodontitis (aOR = 2.25; 95% CI: 1.38–3.66; *p* = 0.004).

Among clinical variables, patients with advanced cancer stage (Stage III/IV) had significantly higher odds of poor periodontal health (aOR = 2.04; 95% CI: 1.03–4.03; *p* = 0.02), as did those with a diagnosis within ≤ 12 months (aOR = 1.42; 95% CI: 1.07–2.18; *p* = 0.03) and those currently undergoing active treatment (aOR = 1.68; 95% CI: 1.03–2.94; *p* = 0.04). Treatment-related variables such as chemotherapy (aOR = 2.21; 95% CI: 1.42–3.56; *p* = 0.001) and radiotherapy (aOR = 1.98; 95% CI: 1.26–3.12; *p* = 0.005) were also significantly associated with breast cancer. However, surgery type (mastectomy vs. lumpectomy) was not statistically significant (aOR = 1.32; 95% CI: 0.88–2.41; *p* = 0.11).

Significant risk factors included age (per 5-year increase) (aOR = 1.17, *p* = 0.003), while protective factors included higher parity (≥ 3 children, aOR = 0.78, *p* = 0.02), younger age at first birth (≤ 30 years, aOR = 0.74, *p* = 0.02), and longer total breastfeeding duration (≥ 36 months, aOR = 0.88, *p* = 0.03). Breast cancer risk was elevated by long-term (≥ 120 months) oral contraceptive use (aOR = 1.54, *p* = 0.02). Socioeconomic status (SES), marital status, last dental visit, BMI, family history, diabetes, smoking, alcohol use, bisphosphonates, and Hormone Replacement Therapy (HRT) were not found to be significantly linked to breast cancer (*p* > 0.05) (Table 3). These findings highlight a significant and independent association between periodontitis severity and breast cancer risk, even after accounting for various demographic, lifestyle, and reproductive factors, underscoring the potential role of oral health in systemic disease processes.

**Table 3:** Multinomial logistic regression with breast cancer as dependent variable

Variables	aOR	95% CI for aOR	*p* value
Periodontitis Severity (Ref: No periodontitis)			
Mild	1.52	0.81–1.92	0.16
Moderate	2.79	1.57–4.89	0.002*
Severe	4.27	2.31–8.26	< 0.001*
Periodontitis Extent (Ref: Localized)			
Generalized	2.25	1.38–3.66	0.004*
Age (per 5- year increase)	1.17	1.06–1.29	0.003*
SES (Ref: Upper)			
Middle	0.94	0.61–1.53	0.86
Lower	1.47	0.69–1.69	0.78
Marital status (Ref: Married)			
Not Married	1.26	0.46–1.61	0.67
Cancer Stage at Diagnosis (Ref: Stage I/II)			
Stage III/IV	2.04	1.03–4.03	0.02*
Time since Diagnosis (Ref: >12 months)			
≤ 12 months	1.42	1.07–2.18	0.03*
Treatment Status (Ref: Post-treatment)			
Active treatment	1.68	1.03–2.94	0.04*
Surgery Type (Ref: Lumpectomy)			
Mastectomy	1.32	0.88–2.41	0.11
Chemotherapy (Ref: No)			
Yes	2.21	1.42–3.56	0.001*
Radiotherapy (Ref: No)			
Yes	1.98	1.26–3.12	0.005*
Last dental visit (Ref: <6 months)			
> 6 months	1.45	0.86–2.37	0.21
BMI (Ref: Normal)			
Underweight	0.94	0.36–1.24	0.82
Overweight	1.28	0.92–1.52	0.12
Obese	1.42	1.11–1.84	0.16
Family history of breast cancer	1.11	0.85–1.46	0.71
Diabetes	1.54	0.42–1.76	0.42
Bisphosphonate use	1.15	0.91–1.32	0.94
Smoking status (Ref: Never)	1.04	0.86–1.26	0.65
Current smoker	2.87	0.78–3.43	0.23
Former smoker	1.41	0.81–1.74	0.61
Alcohol use	1.25	0.68–1.84	0.68
Parity (Ref: No children)			
1–2 children	0.83	0.64–0.94	0.03*
≥ 3 children	0.78	0.68–0.88	0.02*
Age at first birth (Ref: >30 years)			
≤ 30 years	0.74	0.62–0.82	0.02*
Total breastfeeding time (Ref: <12 months)			
12–36 months	0.93	0.76–0.98	0.04*
≥ 36 months	0.88	0.68–0.92	0.03*
Oral contraceptive use (Ref: No use)			
≥ 1-<120 months	1.54	1.42–1.76	0.02*
Time since menopause (Ref: No menopause)			
≥ 1-<60 months	1.15	0.91–1.22	0.94
> 60-<120 months	1.36	0.84–1.54	0.88
Hormone replacement therapy	1.04	0.86–1.26	0.65
Frequency of brushing (Ref: Everyday)			
More than once a day	0.55	0.38–1.82	0.36

*aOR* Adjusted Odds Ratio, *CI* Confidence Interval, *Ref* Reference

*Statistically significant at *p* < 0.05

## Discussion

As evidenced by this study, there is a strong association between periodontitis and breast cancer, with individuals with the disease having worse periodontal health than controls. According to previous literature [17–21], systemic inflammatory pathways, immunological dysregulation, and microbial translocation may all have a role in the development of cancer. The findings contribute to the expanding corpus of research examining the possible connection between dental health and systemic illnesses, including the etiology of cancer.

Even after controlling for potential confounders, the study discovered a strong association between moderate and severe periodontitis and an elevated risk of breast cancer. The risk was 2.79 times greater for women with moderate periodontitis and 4.27 times higher for women with severe periodontitis. According to these results, there is a dose-response association between the degree of periodontitis and the risk of breast cancer. Moreover, the extent of periodontal involvement also played a role; generalized periodontitis was associated with more than twice the odds of breast cancer compared to localized disease. These findings align with the hypothesis that chronic, systemic inflammation originating from widespread periodontal infection may contribute to carcinogenesis through immune dysregulation, microbial translocation, and cytokine-mediated pathways. Comparable results were observed in two studies [3, 22]. 

The study also showed that women with advanced-stage cancer (Stage III/IV), those diagnosed within the past 12 months, and those undergoing active treatment had worse periodontal profiles, possibly reflecting the impact of cancer progression and treatment-induced immunosuppression on oral health. Chemotherapy and radiotherapy were both strongly associated with breast cancer in the model and may act as mediators exacerbating periodontal breakdown by compromising host defense mechanisms, altering the oral microbiome, and reducing salivary flow.

Breast cancer was substantially correlated with older age which was in line with previous literature [17, 23]. Furthermore, this study verified known reproductive and hormonal risk factors. Significantly higher nulliparity, later age at first birth, shorter breastfeeding duration, prolonged oral contraceptive use, and hormone replacement therapy use were significantly more common among breast cancer cases, which are linked to established risk factors associated with prolonged estrogen exposure. These findings were in line with other studies [3, 22]. A number of factors, including socioeconomic status, marital status, surgery type, last dental visit, BMI, family history, diabetes, smoking, alcohol use, bisphosphonates, and hormone replacement therapy (HRT), were not significantly associated with breast cancer (*p* > 0.05), despite the strong association between periodontitis and breast cancer. This demonstrates the intricacy of the etiology of breast cancer and implies that, although persistent oral inflammation might be involved, additional research is necessary to determine other contributing components.

Significantly poorer periodontal health was observed among breast cancer cases, with higher plaque index, gingival index, bleeding on probing, probing depth, clinical attachment loss, and gingival recession compared to controls, suggests that systemic inflammation induced by periodontitis may contribute to breast cancer risk, supporting existing evidence that chronic oral inflammation can impact systemic disease.

Our results are in line with earlier studies that looked at the connection between breast cancer and periodontal disease. Similar to our findings, Sfreddo et al.‘s [3] hospital-based case-control study in Brazil revealed that patients with breast cancer had considerably greater levels of clinical attachment loss, and that periodontitis increased the risk of breast cancer by two to three times. Söder et al. [7] found a strong association between periodontal disease, tooth loss, and breast cancer, further supporting the inflammatory hypothesis.

According to Freudenheim et al., [6] postmenopausal women who self-reported having periodontal disease were 14% more likely to develop breast cancer, especially if they had previously smoked. The Women’s Health Initiative study of postmenopausal women [24] discovered a connection between breast cancer risk and periodontal disease (PD), especially among smokers who had stopped within the previous 20 years, but not for nonsmokers. In their 2009 study of 58 postmenopausal women [29 breast cancer patients on aromatase inhibitors (AI) versus 29 controls], Taichman et al. [22] found that AI users had significantly worse periodontal health, including higher levels of osteocalcin and TNF, attachment loss (*P* < 0.01), and bleeding on probing (*P* = 0.02). These findings suggested a connection between AI therapy and worsening periodontitis.

In contrast, Hujoel et al., [25] used data from the NHANES Epidemiology Follow-up Study and found no association between gingivitis, periodontitis, or tooth loss and breast cancer. Similarly, Mai et al. [8] found no significant association between periodontal disease and breast cancer in postmenopausal women.

These contradictory findings may be due to differences in study design, sample size, periodontal assessment methods, and adjustment for confounders. In contrast to studies that relied on self-reported periodontal disease, our study used clinical periodontal assessments, strengthening the validity of the findings.

Several hypotheses have been offered to explain the putative link between periodontitis and cancer, but the underlying molecular pathways remain unknown. The most widely accepted scientific explanation is that persistent periodontal infection and inflammation can cause a systemic chronic inflammatory state, perhaps acting as a protumor at distant sites [3, 7, 26]. The link between periodontitis and breast cancer may be explained by chronic systemic inflammation and microbial dysbiosis. Pathogens like Porphyromonas gingivalis and Treponema denticola release lipopolysaccharides that elevate cytokines (IL-1β, IL-6, TNF-α, CRP), promoting angiogenesis, DNA damage, and immune evasion. In postmenopausal women, hormonal pathways may also be affected. Oral pathogens can alter distant tissue environments, further supporting the inflammation–cancer connection observed in this study [3, 26, 27]. 

The study has many strengths. One of its main advantages is the use of WHO Oral Health Assessment Form (2013) and validated periodontal indices (Plaque Index, Gingival Index, Probing Depth, Clinical Attachment Loss, and Gingival Recession) to ensure objective and repeatable data collection. It also used Multivariate Analysis for confounders to increase the robustness of the results, the statistical model accounted for a number of potential confounders, such as breastfeeding, diabetes, smoking, alcohol use, BMI, and dental hygiene practices. Selection bias was reduced by carefully matching the controls and cases based on age and tobacco use.

The study has its limitations. Foremost, temporal ambiguity inherent to the cross-sectional design remains a key limitation, which can only be addressed by future prospective longitudinal studies. It remains unclear whether periodontitis preceded the onset of breast cancer or developed as a consequence of the disease or its treatment. This distinction is important, as oncologic therapies particularly chemotherapy and radiotherapy can impair immune function and oral hygiene, potentially worsening periodontal health. Although both treatments were included as covariates in the multivariate regression model to adjust for confounding, residual confounding cannot be ruled out. Second, the small sample size (*N* = 60) reduces statistical power and limits the generalizability of the findings. While this was designed as a pilot study, larger samples are needed to draw more robust conclusions. Third, unmeasured confounders such as stress, genetic predisposition, and dietary factors were not accounted for and may influence both breast cancer risk and periodontal status. Fourth, recall bias may have affected self-reported data on contraceptive use, breastfeeding duration, and oral hygiene behaviors. Finally, the use of convenience sampling may introduce selection bias. Participants were recruited from a single tertiary care center, and the sample may not be representative of the broader population. This limits the generalizability of the findings and may influence the observed association between periodontitis and breast cancer. To confirm these findings, larger, multi-center investigations across India are required.

Given the widespread prevalence of both breast cancer and periodontitis, the findings of this study have important public health implications. If further validated by larger, longitudinal studies, the observed association supports the integration of oral health into broader non-communicable disease (NCD) prevention frameworks. Periodontal screening could be considered as a component of routine health assessments in women, especially those with known reproductive or lifestyle risk factors for breast cancer.

Promoting oral health awareness, especially among women at risk for or living with breast cancer, may offer an additional avenue for early intervention and risk reduction. Early identification and management of periodontal disease may offer a low-cost, non-invasive strategy to mitigate systemic inflammation, potentially reducing the risk or severity of breast cancer. Public health programs targeting women’s health particularly in low- and middle-income countries like India could benefit from interdisciplinary collaboration between dental and medical professionals. Incorporating periodontal assessment into cancer prevention and survivorship guidelines may improve overall health outcomes and promote holistic, preventive care. These findings advocate for policy-level recognition of oral health as a vital part of women’s health strategies.

## Conclusion

There is a significant association between periodontitis and breast cancer as evidenced in this study. A higher risk of breast cancer was independently linked to severe periodontitis, and women with breast cancer had far worse periodontal health. These results imply that long-term periodontal inflammation might have a role in the development of breast cancer. The cross-sectional design of the study precludes drawing conclusions about causality, but it does draw attention to the need for more longitudinal studies. The findings of this study underscore the importance of oral health in cancer prevention and warrant further studies in larger populations.

## Data Availability

No datasets were generated or analysed during the current study.
